# End-of-life care in low- and middle-income countries

**DOI:** 10.2471/BLT.16.185199

**Published:** 2017-11-01

**Authors:** Dulce M Cruz-Oliver, Milta O Little, Jean Woo, John E Morley

**Affiliations:** a Division of Geriatric Medicine, Saint Louis University School of Medicine, 1402 S. Grand Blvd., M238, Saint Louis, MO, 63104, United States of America.; b Department of Medicine and Therapeutics, The Chinese University of Hong Kong, Hong Kong, China.

Health-care providers are recognizing the importance of palliative and end-of-life care as a treatment option for seriously ill patients. Palliative care is an approach to improve the quality of life of patients and their families facing problems associated with life-threatening illness, through the prevention and relief of suffering, by means of early identification, assessment and treatment of pain and other distresses.^1^ Palliative care begins at the start of a serious illness and is given alongside treatments designed to combat the disease. End-of-life care is a type of palliative care for people in the final months of life and is considered when the person’s condition deteriorates and active treatment does not control the disease. Palliative and end-of-life care helps those with advanced, progressive, incurable and serious illness to live as well as possible until they die.^2^

In 2014, the World Health Organization (WHO) in collaboration with the Worldwide Palliative Care Alliance, published the *Global atlas of palliative care at the end of life*.^1^ The document presented a global estimate on the availability of palliative care and provided answers to nine core questions, including what palliative care is, why it is a human right and what’s needed to advance the field. The report highlighted that palliative-care need should be defined based on the needs of individuals and not on their diagnosis or prognosis. Providing adequate palliative care and relief of unnecessary suffering to all people and specially to those most vulnerable in society is a human right.^1^^,^^3^ However, despite this recognition, there are still many low- and middle-income countries that lack palliative-care services. Worldwide, an estimated 20 million people require palliative care every year. Most of those who need palliative care are older adults (69%) and 78% of them live in low- and middle-income countries.^1^

The report also showed that palliative-care services in low- and middle-income countries are not integrated into national health systems, limiting the availability of end-of-life care in countries that need it most. The lack of progress in integration has been attributed to misinformation on what palliative care-related services provide, lack of financial support to sustain such programmes and cultural beliefs around death.^1^^,^^4^^,^^5^ Such beliefs and knowledge gaps hindered progress in many countries to either establish or increase access to palliative and end-of-life care services and to work on legislation that regulates opioid prescription by clinicians and their use by patients.^1^^,^^4^^,^^5^

The report highlighted that globally, there are limited guidelines for establishing palliative-care programmes and their availability in low- and middle-income countries is also unknown. Where they exist, the guidelines are often in English and the different translations that are available are mostly in five languages – Bengali, French, Mandarin, Portuguese and Spanish – which limit their full use in many underserved areas.^1^ Regional models for palliative-care programmes that can be locally adapted and funded need to be developed.

To scale-up palliative-care services, the report proposes the use of a population health model to develop palliative-care programmes.^1^ The focus of palliative care is to achieve patient-centred outcomes, such as improvement in quality of life and emotional and spiritual well-being while also reducing the burden on caregivers and providing support to family caregivers during bereavement. Population health on the other hand, focuses on improving morbidity, mortality and reducing health disparities at the population level. Applying a population health model will move the field of palliative care away from the current focus on inpatient and outpatient consultation to one that focuses on specific populations, such as children, older people and vulnerable populations.^1^^,^^6^
